# BACE1: More than just a β‐secretase

**DOI:** 10.1111/obr.13430

**Published:** 2022-02-04

**Authors:** Hannah A. Taylor, Lena Przemylska, Eva M. Clavane, Paul J. Meakin

**Affiliations:** ^1^ Discovery & Translational Science Department, Leeds Institute of Cardiovascular and Metabolic Medicine University of Leeds Leeds UK

**Keywords:** BACE1, obesity, type 2 diabetes

## Abstract

β‐site amyloid precursor protein cleaving enzyme‐1 (BACE1) research has historically focused on its actions as the β‐secretase responsible for the production of β‐amyloid beta, observed in Alzheimer's disease. Although the greatest expression of BACE1 is found in the brain, BACE1 mRNA and protein is also found in many cell types including pancreatic β‐cells, adipocytes, hepatocytes, and vascular cells. Pathologically elevated BACE1 expression in these cells has been implicated in the development of metabolic diseases, including type 2 diabetes, obesity, and cardiovascular disease. In this review, we examine key questions surrounding the BACE1 literature, including how is BACE1 regulated and how dysregulation may occur in disease, and understand how BACE1 regulates metabolism via cleavage of a myriad of substrates. The phenotype of the BACE1 knockout mice models, including reduced weight gain, increased energy expenditure, and enhanced leptin signaling, proposes a physiological role of BACE1 in regulating energy metabolism and homeostasis. Taken together with the weight loss observed with BACE1 inhibitors in clinical trials, these data highlight a novel role for BACE1 in regulation of metabolic physiology. Finally, this review aims to examine the possibility that BACE1 inhibitors could provide a innovative treatment for obesity and its comorbidities.

## Graphical abstract

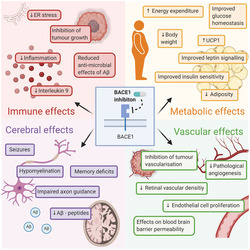

## 1 INTRODUCTION

Beta‐site amyloid precursor protein cleaving enzyme‐1 (BACE1) research has historically focused on the brain and its actions as the β‐secretase responsible for the production of amyloid beta (Aβ) peptides. These Aβ peptides accumulate into senile plaques, characteristic of Alzheimer's disease (AD), and cause neuronal death and cognitive decline.^1^ Although the greatest expression of BACE1 is found in the brain and pancreas, BACE1 mRNA and protein is also found at low levels in many cell types.^2^, ^3^ Pathologically, BACE1 has also been implicated in the development of other diseases, including type 2 diabetes, schizophrenia, and epilepsy.^4^, ^5^ The phenotype of the BACE1 knockout mice models, including reduced weight gain, hypomyelination, and associations with metabolic diseases such as diabetes, proposes a physiological role of BACE1 in energy metabolism and homeostasis.^6^ Together with the effects observed with BACE1 inhibitors in clinical trials, this highlights the lack of understanding around the physiological functions of BACE1. In this review we explore the dysfunction, and physiological functions of BACE1, including its regulation, and role in energy metabolism and homeostasis.

## 2 BACE1 STRUCTURE

**FIGURE 1:**
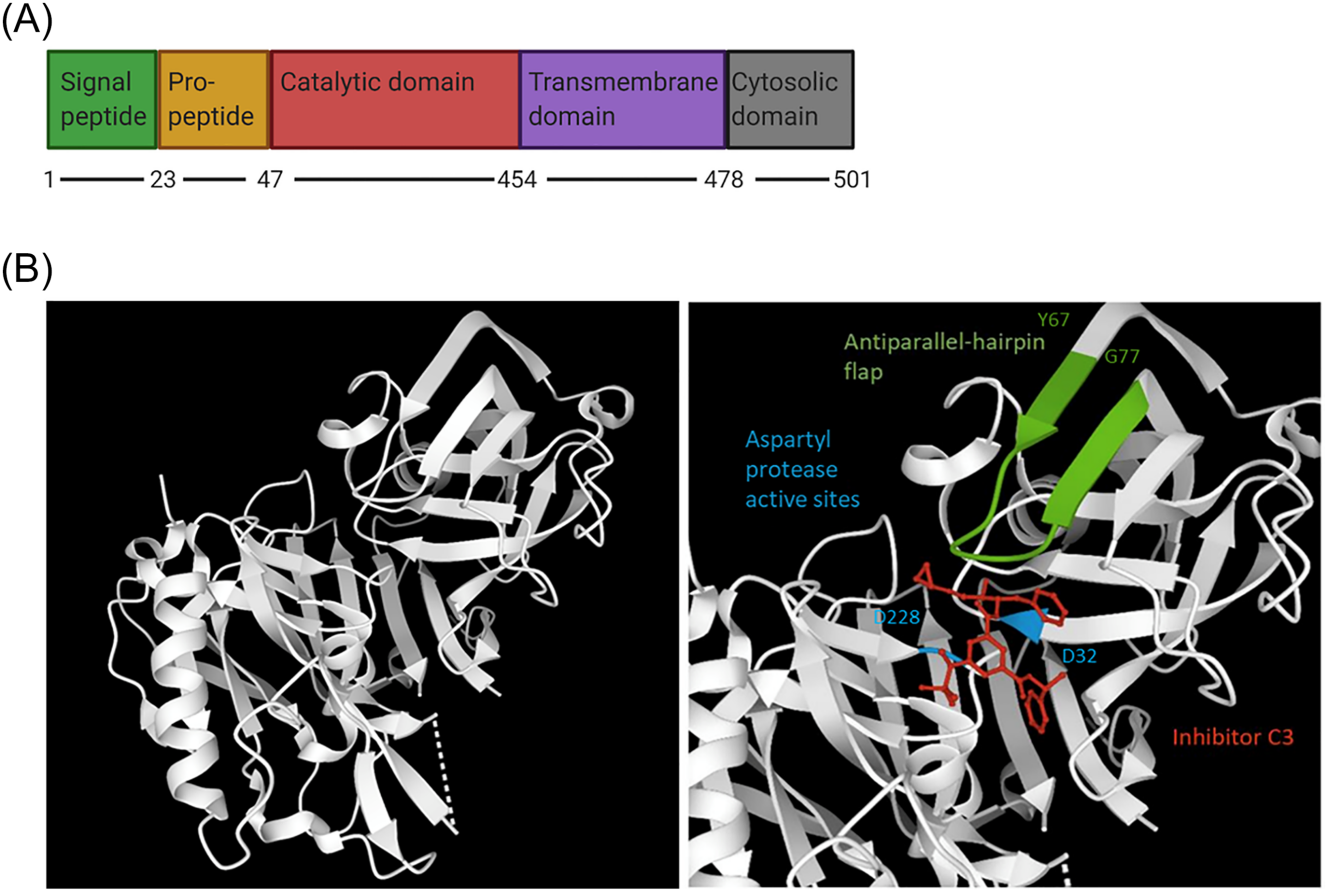
The structure and domains of BACE1. (A) The primary structure of BACE1 with functional domains labeled. There are five domains: signal peptide (1–23), pro‐peptide (23–47), catalytic domain (47–454), transmembrane domain (454–478), and the cytosolic domain (478–501). Created with BioRender.com. (B) BACE1 crystal structure made using structure 3TPR on RSCB Protein DataBank (https://www.rcsb.org/). The unannotated structure (left) and inhibitor C3 binding (red), the two aspartyl protease active sites (blue) at D93 and D289, and the antiparallel‐hairpin flap (green) between Y128‐G138, are shown (right). The structure is missing 62 residues

BACE1 is a type 1 membrane protein which, together with BACE2, forms a subfamily of membrane anchored aspartyl proteases.^7^ The BACE1 gene is transcribed as a 501 amino acid preprotein, containing five key domains, a signal peptide and pro‐, catalytic, transmembrane, and cytoplasmic domains (Figure 1A). The signal peptide traffics the BACE1 preprotein to the endoplasmic reticulum (ER), where furin cleavage of the pro‐domain produces a mature BACE1 protein.^6^ The transmembrane domain determines BACE1 localization to the late Golgi, where in the trans‐Golgi network BACE1 is post‐translationally activated. The protease activity of BACE1 is dependent on two aspartyl active sites at D93 and D289, as well as the position of the regulatory antiparallel‐hairpin flap relative to the substrate binding site (Figure 1B).^8^ The activated BACE1 protein subsequently operates at the plasma membrane in endosomes and the Golgi apparatus, functioning at an optimal pH of 4.5.^8^

## 3 ESTABLISHED BACE1 SUBSTRATES AND FUNCTIONS

Most knowledge and research around BACE1 is focused on its role in the amyloidogenic pathway, where it is responsible for the initial rate limiting cleavage of the APP protein. Sequential cleavage by BACE1 and γ‐secretase produces Aβ‐40 and Aβ‐42 peptides. Despite having around 64% homology to BACE1, BACE2 cleaves the APP protein at an alternative site and therefore does not elicit the same β‐secretase action and Aβ production.^9^ BACE2 is also mainly found in peripheral tissues, in contrast to BACE1 which, by comparison, is highly expressed in the brain.^10^ BACE1 production of Aβ‐42 is associated with regulation of memory, synaptic function, myelin repair, and AD.^5^ AD is the most common form of dementia and presents with several physiological changes in addition to the accumulation of Aβ‐42 into extracellular amyloid plaques, including neurofibrillary tangles (NFTs), chronic inflammation, synapse loss, neuronal death, and hypometabolism.^11^, ^12^ The accumulation Aβ‐plaques disrupts neuronal and synaptic functions leading to detrimental cognitive effects.^5^ However, there is increasing evidence for non‐neuronal functions of Aβ and consequences of altered BACE1 activity. The increased production of Aβ has been linked to cerebrovascular impairments including capillary degradation, impairments in the blood brain barrier (BBB), and response to vascular injury.^13^, ^14^ Some of these effects may be linked to the antimicrobial role of Aβ and its release into the blood from activated platelets in response to inflammation and immune responses.^15^

Furthermore, Aβ has been shown to regulate transcription of APP and insulin‐like growth factor receptors,^16^ and BACE1 has been implicated in a range of metabolic functions.^17^, ^18^, ^19^ This suggests various important physiological roles of BACE1 in different cells and organelles, in addition to its role in AD. Although the expression of BACE1 is highest in the brain, it is found widely expressed at lower levels in other tissues including endocrine tissue, the pancreas, muscle tissue, respiratory tissue, bone marrow, and lymphoid tissue.^20^, ^21^ As APP is also widely expressed, the BACE1‐mediated production of Aβ could have an effect on many cells and tissues.^20^, ^21^

It is well acknowledged that APP is a poor substrate for BACE1 and, through secretome enrichment experiments, nearly 70 BACE1 substrates have been identified to date.^22^, ^23^, ^24^, ^25^ Many BACE1 substrates, like APP, are type 1 membrane proteins whose function can be enhanced or reduced following BACE1‐mediated shedding.

Neuregulin 1 (NRG1) is a signaling protein involved in various cellular functions, including cell growth and differentiation. BACE1 cleavage of NRG1 regulates myelination,^26^, ^27^ and increased BACE1 cleavage of NRG1 has been implicated in the development of schizophrenia.^28^ BACE1 cleavage of Jagged 1 (Jag1) regulates the Jag1‐Notch signaling pathway important in the control of astrogenesis and neurogenesis.^29^ Seizure protein 6 (SEZ6) and seizure‐like protein 6 (SEZ6L) proteins influence ER functions in neurons, control synaptic connectivity, and motor coordination and therefore may likely be responsible for the seizures, motor deficits, and reduced spinal deficiency observed in BACE1 null mice.^24^ Cache domain containing protein‐1 (CACHD1) and neural cell adhesion molecules (NCAM1 and 2) are involved in synapse formation, maturation, and maintenance.^22^, ^30^ L1 cell adhesion molecule (L1CAM) and neural cell adhesion molecule L1‐like protein (CHL1) are BACE1 substrates involved in axon guidance.^31^ Substrates SEZ6, L1CAM, leucine rich repeat neuronal 1 (LRRN1), neurotrimin, and CHL1 are all involved in neurite outgrowth.^23^ BACE1 has also been implicated in regulation of sodium channel metabolism in neuronal cells, via its cleavage of voltage‐gated sodium channel β2 subunit (Navβ2).^32^

To date, the BACE1 substrates identified are primarily associated with neurological function and the central nervous system. However, this might be more representative of experimental designs. Therefore, the true extent of non‐CNS functions may not yet be fully known.

## 4 NON‐NEURONAL BACE1 SUBSTRATES

The phenotype of the BACE1 knockout mice suggests BACE1 plays additional roles to regulation of neuronal function. Accordingly, non‐neuronal physiological effects of BACE1 have come to light recently. P‐selectin glycoprotein ligand‐1 (PSGL‐1) is a BACE1 substrate that plays an important role in immune defenses by recruiting white blood cells to the site of infection.^25^ Another BACE1 substrate, interleukin‐1 receptor II (IL‐1R2), an interleukin‐1 decoy receptor, presents a mechanism for abnormal inflammation in response to changes in BACE1 activity.^33^ It is therefore evident that BACE1 is involved in many pathways and processes, and its effects on a single protein can have a complex cascade effect on other functions of the body. Through these additional substrates, BACE1 has been implicated in inflammation, cardiovascular function, glucose homeostasis, and insulin signaling^18^, ^34^, ^35^, ^36^, ^37^, ^38^ (Table 1). This highlights the importance of investigating all substrates and pathways BACE1 is involved in, both to understand implications of its dysfunction and to benefit the development of effective therapeutics.

**TABLE 1:** BACE1 substrates involved in non‐neuronal physiological functions

Gene	Protein	Physiological function	Experimental evidence
APP	Amyloid precursor protein	Aβ contributes to vascular impairments, inflammation and insulin resistance. sAPPβ causes ER stress, inflammation and insulin resistance	Aβ and APP overexpression induce endothelial dysfunction.^37^ Aβ levels in human plasma correlate with vascular function and diabetes.^39^ sAPPβ administration mimics palmitate induced ER stress, inflammation and insulin resistance in skeletal muscle and adipose tissue, which is reduced with BACE1 inhibition.^40^
IL‐1R2	Interleukin‐1 receptor I	Regulates inflammatory and immunoregulatory cytokine IL‐1 function through NFκB signaling.	Increased BACE1 expression in in vitro models show increased levels of IL‐1R2 shedding. Soluble IL‐1R2 is released into the circulation and modulates systemic IL1 activity.^33^
Jag1	Jagged 1 protein	Plays role in hematopoiesis and cardiovascular development through interactions with Notch 1 receptors.	BACE1 cleavage sites mapping and site‐directed mutagenesis assays, confirmed Jag1 as BACE1 Substrate.^41^ The function of the soluble Jagged 1 ectodomain is unclear, however most likely to antagonize Notch signaling.
Jag2	Jagged 2 protein	Homolog of Jag1, also involved in Notch signaling.	Jag 2 was less effectively cleaved by BACE1 but also confirmed as its substrate.^41^ Functions similar to Jag1.
PSGL‐1	P‐selectin glycoprotein ligand‐1	Involved in mediating leukocyte adhesion to endothelial cells during inflammation and tissue injury.	Cleavage site of PSGL‐1 by BACE1 was mapped through deletion constructs and enzymatic deglycosylation of the C‐terminal PSGL‐1 fragment.^25^ Soluble PSGL‐1 may disrupt leukocyte adhesion and diapedesis
ST6Gal 1	α2,6‐Sialyltransferase	Terminal step of N‐glycan biosynthesis of glycoproteins and plays a role in formation of atherosclerosis	In vitro experiments show that STGal 1 is cleaved by BACE1 between Leu37 and Gln38.^42^ Soluble ST6Gal1 may disrupt leukocyte adhesion and diapedesis
APLP1 and APLP2	Amyloid beta precursor‐like protein 1 and 2	Regulate glucose and insulin homeostasis	In vivo experiments show that APLP knockout mice show the same AD‐like symptoms that APP knockout mice experience.^43^ APLP knockout mice display improved glucose clearance and insulin production.^44^
IR	Insulin receptor	Regulates glucose homeostasis and insulin sensitivity	IR is cleaved by BACE1, producing IRsol and reducing functional cell surface IR. Cleavage has been confirmed in both in vitro and in vivo experiments.^18^
VEGFR1 (Flt‐1)	Vascular endothelial growth factor receptor 1	Decoy receptor for VEGF signaling, negatively regulates angiogenesis	BACE1 knockout mice have retinal pathology owing to reduced sFlt‐1 and enhanced angiogenesis.^45^ Soluble Flt‐1 plays a role in termination of a pregnancy.

*Note*: This table shows confirmed BACE1 substrates with physiological roles, their functions, and experimental evidence.

## 5 CONSEQUENCES OF CHANGES IN BACE1 EXPRESSION AND ACTIVITY

Numerous physiological effects are observed in response to changes in BACE1 expression and activity in disease models, and potential therapeutics. In a variety of pathological conditions, including AD, cerebral amyloid angiopathy, and metabolic diseases such as type 2 diabetes and obesity, BACE1 expression and activity are increased and drive disease progression.^3^, ^34^, ^39^, ^40^ Accordingly, BACE1 knock‐in mice display an AD‐like pathology including elevated levels of Aβ plaques, synaptic impairments, decreased cognitive function,^41^ and systemic diabetes.^34^ Conversely, BACE1 knockout mice display reduced birth weight, hypomyelination, memory deficits, behavioral alterations, axon guidance impairment, impaired midbrain dopaminergic signaling, seizures, and abnormal electroencephalograms (EEGs).^42^, ^43^ Some of the observed phenotypes, such as impaired axon guidance, are more severe when BACE1 is deficient in the developmental stages.^43^

In addition to its neuronal roles, changes in BACE1 activity have been associated with physiological functions including maintenance of the blood–brain barrier (BBB), angiogenesis, protection against obesity, immune and antimicrobial properties, inflammatory response, and tumor suppression.^44^, ^45^, ^46^, ^47^ This proposes a metabolic function of BACE1, and further research into BACE1 and its substrates is unveiling further physiological functions, which will be further discussed below.

## 6 REGULATION OF BACE1 AT THE TRANSCRIPTIONAL, TRANSLATIONAL, AND POST‐TRANSLATIONAL LEVEL

BACE1 activity is regulated at the transcriptional, post‐transcriptional, and translation level, with post‐translational modifications (PTMs) required to produce the mature BACE1 protein.^48^ At the transcriptional level, BACE1 is tightly regulated by its promoter region, which contains numerous transcriptional binding sites. Of specific interest to this review are the metabolically regulated transcription factors hypoxia‐inducible factor‐1 (HIF‐1), cAMP‐response element binding protein (CREB), signaling transducer and activator of transcription 1 (STAT1), and peroxisome proliferator‐activated receptor gamma (PPARy).^1^ BACE1 levels and activity increase in response to hypoxia, energy disruption, and mitochondrial stress, owing to HIF‐1 binding the BACE1 promoter.^49^ Hypoxia causes the hypoxia‐responsive element (HRE) to bind the HIF‐1 transcription factor, stimulating gene activation. Overexpression of BACE1 has been shown to decrease CREB phosphorylation, in turn decreasing protein kinase A (PKA) activity, and cyclic adenosine monophosphate (cAMP) levels.^50^ Although not well characterized, the presence of a CREB binding site within the BACE1 promoter suggests that BACE1 regulation of the cAMP/PKA/CREB pathway, significant in glucose metabolism and lipid homeostasis, may feedback to regulation of BACE1 gene expression.^51^

Inflammation is closely associated with metabolic disease, and increases in BACE1 expression in response to inflammation have been attributed to transcriptional regulation. The pro‐inflammatory cytokine interferon‐gamma (IFNy) causes an increase in BACE1 through subsequent Janus Kinase 2 (JAK2) and mitogen activated protein (MAP) kinase signaling causing phosphorylation of STAT1.^52^ The phosphorylation of STAT1 leads to its binding of the BACE1 promoter, increasing BACE1 gene expression.^52^ This action of STAT1 can be inhibited by suppressor of cytokine signaling (SOCS) 1 and 3, which prevent phosphorylation of STAT1 on the Tyrosine 701 residue.^52^ Additionally, studies showed that NSAIDs, used to decrease inflammation, lower BACE1 activity, likely by reducing PPARy levels.^53^, ^54^ PPARy is a transcriptional regulator, which acts upon the BACE1 promoter increasing gene expression. Regulation of expression by transcription factors is therefore an important factor in the physiological function of BACE1, as well as its dysregulation in disease.

**FIGURE 2:**
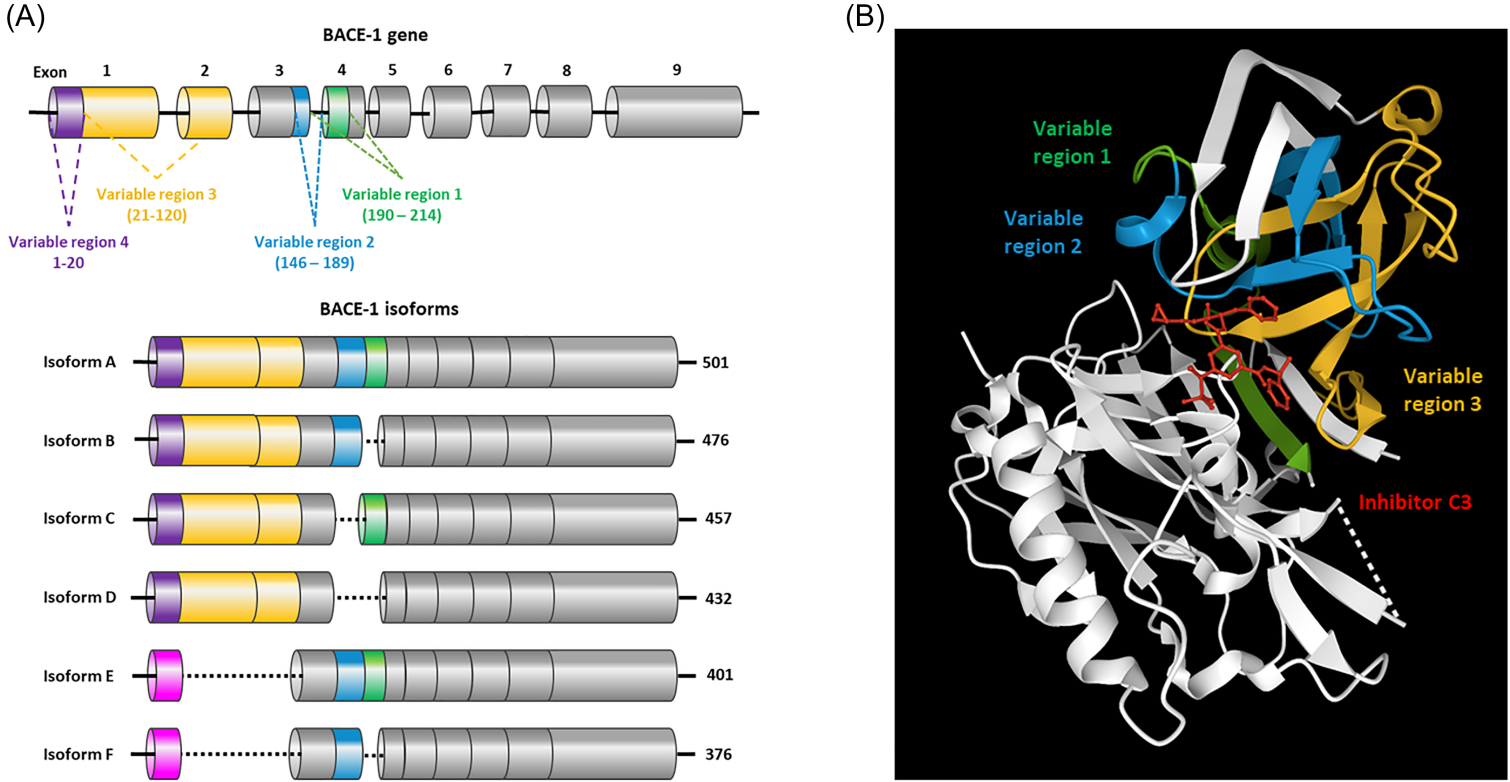
Alternative splicing of BACE1 produces different isoforms. (A) BACE1 is alternatively spliced at four variable regions; 190–214 (green), 146–189 (blue), 21–120 (yellow), and 1–20 (purple). Alternative splicing of these regions produces at least six distinct isoforms, the most characterized being Isoforms A–F: Isoform A (501aa), Isoform B (476aa), Isoform C (457aa), Isoform D (432aa), Isoform E (401aa), Isoform F (376aa) are depicted. Isoforms E and F contain an alternative Exon 1 (pink). (B) The variable regions shown on the BACE1 crystal structure with inhibitor C3 (red) made using structure 3TPR, on RSCB Protein DataBank (https://www.rcsb.org/). Variable regions 1 (190–214) (green), 2 (146–189) (blue), and 3 (21–120) (yellow) show close proximity to the inhibitor binding site. The structure is missing 62 residues, including variable region 1–20, which therefore could not be labeled

In addition to regulation by transcription factors, BACE1 gene expression is positively regulated at the transcriptional level by the BACE1 antisense transcript (BACE1‐AS).^55^ BACE1‐AS is reported to stabilize BACE1 through competitive binding of miR‐485‐5p which would otherwise repress BACE1 mRNA translation.^56^ It is therefore possible that, by acting as a sponge, BACE1‐AS regulates other miRNAs in a similar manner, significant as numerous miRNAs are reported to repress BACE1.^57^, ^58^, ^59^, ^60^, ^61^, ^62^, ^63^ The action of BACE1‐AS has been implicated in AD pathology, with increased BACE‐AS in turn increasing BACE1 and Aβ‐42.^64^ Dysregulation of the BACE1/BACE1‐AS axis also has been implicated in both cardiac dysfunction and epilepsy.^38^, ^65^ The BACE1 mRNA transcript is further regulated by alternative splicing, reported to influence the activity of the resulting protein. There are six relatively established BACE1 isoforms (A–F), in addition to reports of various less well‐characterized forms including BACE1‐455 and BACE1‐127^66^, ^67^, ^68^, ^69^ (Figure 2). Isoform A is 501 amino acids long and is reported to have superior protease activity.^68^ Although BACE1 isoforms B–F are reported to have reduced A□ producing activity in comparison with isoform A, their relative levels of protease activity are less well defined. The different isoforms have been reported in varying abundance in different tissues and ages.^70^, ^71^ This suggests that alternative splicing may be an important mechanism of regulation of BACE1 activity and may underlie cleavage of different substrates and tissue‐specific effects; however, this requires further investigation.

**FIGURE 3:**
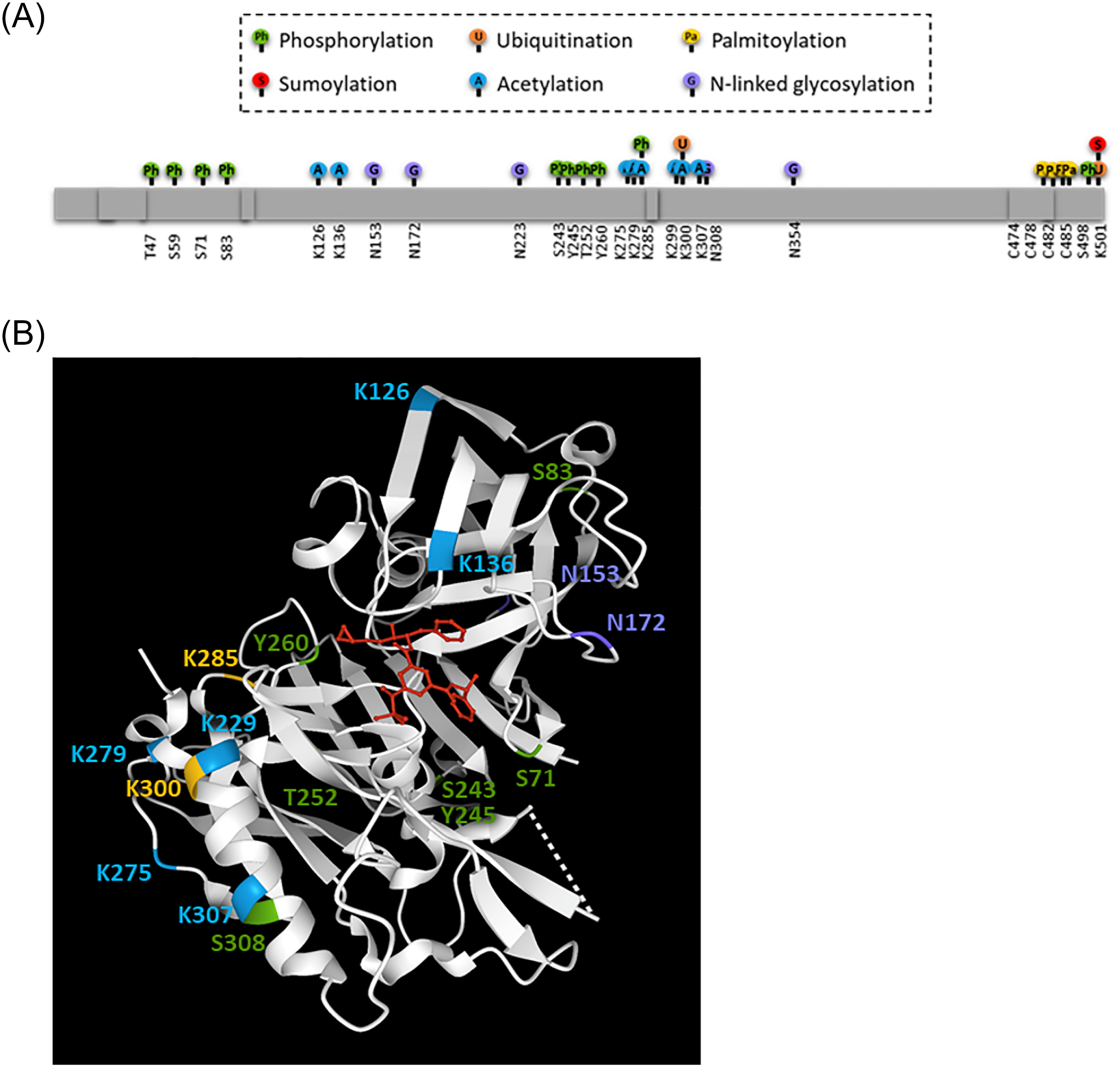
Post‐translational modifications on BACE1. (A) Diagram showing all post‐translational modifications reported in the literature, and predicted phosphorylation sites from Phosphosite (https://www.phosphosite.org/). This includes phosphorylation (green) at T47, S59, S71, S83, S243, S245, S252, S260, S308, and S498. SUMOylation (red) at K501. Ubiquitination (orange) at K285, K300 and K501. Acetylation (blue) at K126, K136, K275, K279, K285, K299, K300, and K307. Palmitoylation (yellow) at C478, C482, C485, and C474. N‐linked glycosylation (purple) at N153, N172, D223, and D354. (B) BACE1 crystal structure with inhibitor C3 (red) made using structure 3TPR on RSCB Protein DataBank (https://www.rcsb.org/). Not all modifications are shown as 62 residues are missing, including N‐ and C‐terminal regions. Phosphorylation (green), ubiquitination (orange), acetylation (blue) and N‐linked glycosylation sites (purple) are labeled on the BACE1 crystal structure

Post‐translationally, BACE1 is subject to numerous modifications that have important effects on trafficking, stability, activity, and degradation^48^, ^72^(Figure 3). Alterations in PTMs are a way of inducing changes in function and activity in response to physiological changes. This is true for BACE1, with various PTMs strongly influencing activity. In order for the mature BACE1 protein to be produced, glycosylation and transient acetylation in the ER are required. This promotes trafficking of the immature protein to the trans‐golgi network, preventing its degradation.^73^ The BACE1 protein has four N‐linked glycosylation sites, which are targeted in the Golgi and facilitate pro‐peptide processing, maturation, and transportation.^74^ Missing glycosylation sites in alternatively spliced isoforms of BACE1 are reported to contribute to reduced secretase activity.^75^ Glycosylation has also been implicated in the pathological increases in BACE1 stability seen in response to oxidative stress, with bisecting N‐acetylglucosamine (GlcNAc), catalyzed by GlcNAc transferase Gnt‐III having been shown to prevent lysosomal degradation of BACE1.^76^, ^77^ BACE1 is also phosphorylated, with phosphorylation at threonine 252 via p25/Cdk5 pathways found to stimulate protease activity.^78^ Another important phosphorylation site is located as serine 498, by casein kinase 1. Phosphorylation is important in intracellular trafficking, recognition by Golgi‐localized γ‐ear‐containing ARF‐binding (GGAs), and retention in acidic compartments when activated, enabling substrate interactions.^79^, ^80^, ^81^ Furthermore obesity increases cdk5 and casein kinase 1 activity is enhanced. The BACE1 protein is also acetylated at seven different lysine residues mediated by CoA:lysine acetyltransferase 1 and 2 (ATase 1 and ATase 2), and ubiquitinated at K501 which causes translocation to the lysosomes for degradation.^6^ The ubiquitination site at 501 is also competitively SUMOylated, which stabilizes the protein and promotes its activity.^72^ Dysregulation of PTMs therefore has a substantial impact on BACE1, either altering enzyme activity or cellular localization, and may play an important role in alterations in BACE1 activity in response to physiological stimuli.^48^

## 7 A METABOLIC ROLE OF BACE1: THE LINK BETWEEN TYPE 2 DIABETES AND AD?

Metabolic dysfunction and type 2 diabetes mellitus (T2D) are long established risk factors for AD, with T2D sufferers having a 50% increased risk of developing AD.^82^ Although the mechanisms underlying this connection are not fully understood, the increasing evidence for a role of BACE1 in metabolism and T2D presents a possible link. The prevalence of T2D is significant, with the World Health Organization (WHO) reporting 422 million cases worldwide in 2014, with this only expected to increase. T2D is caused by decreased sensitivity to insulin, leading to hyperglycemia which can cause serious damage especially to nerves and blood vessels. Recent studies implicate BACE1 in alterations in insulin and leptin signaling, presenting BACE1 action as a potential link between AD and T2D.^83^ BACE1 has been implicated in T2D via Aβ‐dependent and independent processes, which will be reviewed below.

## 8 BACE1 REGULATION OF INSULIN PATHWAYS

BACE1 is implicated in the pathogenesis of T2D through the insulin pathway. Ordinarily, high blood glucose levels stimulate insulin production in the pancreas and release into the bloodstream, where it then travels to insulin responsive tissues such as skeletal muscle, liver, and adipose tissue.^84^ Cells detect increased levels of insulin through insulin receptors, which then stimulates a phosphorylation cascade.^85^ This leads to tissue‐specific effects such as prevention of lipolysis, glucose uptake, stimulation of glycogenesis and lipogenesis, promotion of protein synthesis, and upregulation of genes such as fatty acid synthase and malic enzyme genes.^84^ Collectively, this lowers blood glucose levels, maintaining homeostasis, and vasodilation.^86^

**FIGURE 4:**
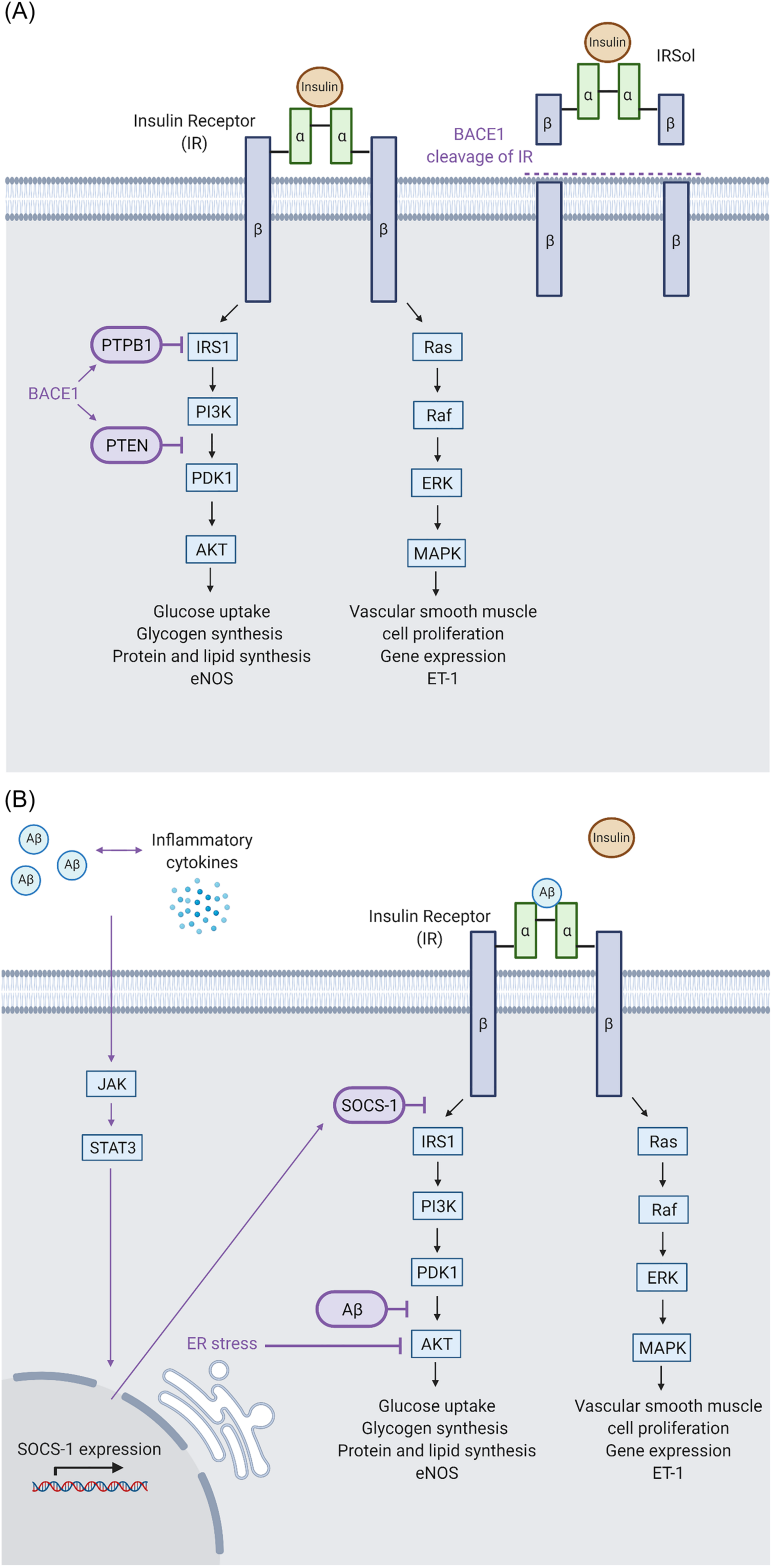
The role of BACE1 in the insulin signaling pathway. (A) BACE1 can impact insulin signaling through the negative regulators of the insulin signaling pathway, PTBP1 and PTEN (left), and through cleavage of the insulin receptor (IR) (right). PTBP1 and PTEN inhibit PI3K/Akt signaling affecting glucose uptake, glycogen synthesis, protein and lipid synthesis, and vasodilation. Cleavage of the insulin receptor prevents signaling in response to insulin binding. (B) BACE1‐mediated Aβ production can also impact insulin signaling. The accumulation of Aβ causes inflammation, which in turn stimulates the JAK/STAT3 signaling cascade, increased SOCS‐1 expression and inhibition of insulin signaling. PI3K/Akt signaling can also be inhibited by ER stress and Aβ mediated interruption of phosphoinositide‐dependent kinase‐1 (PDK) activity through binding with its target protein kinase B (PKB/Akt). Additionally, Aβ competitively binds the insulin receptor (IR) preventing insulin binding. Elevated BACE1 activity and Aβ can therefore lead to dysregulated insulin signaling. Created with BioRender.com

BACE1 plays a pivotal role in effective insulin signaling, with high levels of BACE1 leading to reduced insulin signaling and glucose uptake, and vice versa. The influence of BACE1 on insulin signaling is in part through negative regulators PTEN and PTP1B, which are found to decrease in response to BACE1 inhibition.^18^, ^19^ Furthermore, BACE1 can reduce the cell surface expression of biologically active IRs via cleavage of its ectodomain, in a glucose‐dependent manner^18^ (Figure 4A). The released soluble IR (IRsol) is able to bind insulin, reducing the abundance of bioavailable insulin, and further impairing insulin signaling. Tissue expression of BACE1 and plasma levels of the IRsol are higher in patients with T2D, supporting the theory that BACE1 cleavage of IR drives the disease.^4^, ^87^ In zebrafish, BACE2 has also been implicated in the negative regulation of insulin signaling through the cleavage and trafficking of the IR.^88^ Furthermore, therapeutic inhibition of BACE2 in mice, has been linked to increased pancreatic β‐cell function and mass, enhanced insulin production and improved glucose homeostasis, likely via its role in the cleavage of Tmem27.^89^

BACE1 and BACE2 activity can modulate insulin sensitivity indirectly via the production of Aβ. APP, BACE1, and BACE2 are all expressed in the liver, and a 30% increase in circulating Aβ was observed when APP expression was restricted to hepatocytes in a mouse model.^90^ The production of Aβ is known to happen in numerous cell types, with platelets being responsible for 90% of circulating Aβ,^91^ and glial cells being a major source of Aβ.^92^ Insulin and Aβ have a similar tertiary structure and both bind to IR, making Aβ a competitive inhibitor able to reduce cellular insulin sensitivity^93^, ^94^ (Figure 4B). Insulin sensitivity is also affected by Aβ‐mediated upregulation of SOCS‐1, an inhibitor of the interferon‐gamma pathway.^95^ SOCS‐1 is upregulated by Aβ via the JAK2/STAT3 pathway, leading to insulin resistance.^95^, ^96^ Notably, JAK/STAT3 signaling is also regulated by leptin, providing a link to other aspects of the metabolic syndrome.^97^ Lee *et al* showed that Aβ causes insulin resistance by interruption of phosphoinositide‐dependent kinase‐1 (PDK) activity through binding with its target protein kinase B (PKB/Akt).^98^ Furthermore, palmitate induced inflammation and ER stress impairs insulin signaling, as observed in obese individuals with T2D. BACE1 inhibition can restore insulin signaling in this setting, while mimicked by sAPPβ.^44^

BACE1 and BACE2 have been shown to be expressed in skeletal muscle, where they have been found in the neuromuscular junctions of normal adult muscle,^99^ and found to colocalize with Aβ in a pathological setting in muscle.^100^ This is notable as around 70%–80% of circulating glucose is transported into skeletal muscle via GLUT4 glucose receptors, which are predominately expressed in muscle and adipose tissue. Muscle glucose transport is impaired in DIO mice,^101^ and transport via GLUT4 receptors is compromised in insulin‐resistant diabetes,^102^ with signaling events downstream of the insulin receptor being linked to trafficking of GLUT4. If BACE1 is expressed at a great enough level to produce pathological levels of Aβ in the skeletal muscle, this suggests it may be present at an abundance able to cleave the IR receptor, and impact insulin signaling both directly through IR cleavage and indirectly through Aβ production, in skeletal muscle. This is supported by the findings that APP processing in C2C12 myotubes directly affects glucose uptake and GLUT4 translocation.^103^ Therefore, as APP and BACE1 are widely expressed, local Aβ production could impact signaling in many cells and tissues.

Taken together, this presents a strong physiological role of BACE1 in insulin signaling. This may have important implications in the context of T2D and obesity, as well as providing a potential mechanistic explanation for the increased risk of AD in T2D patients.

## 9 LEPTIN PATHWAY

**FIGURE 5:**
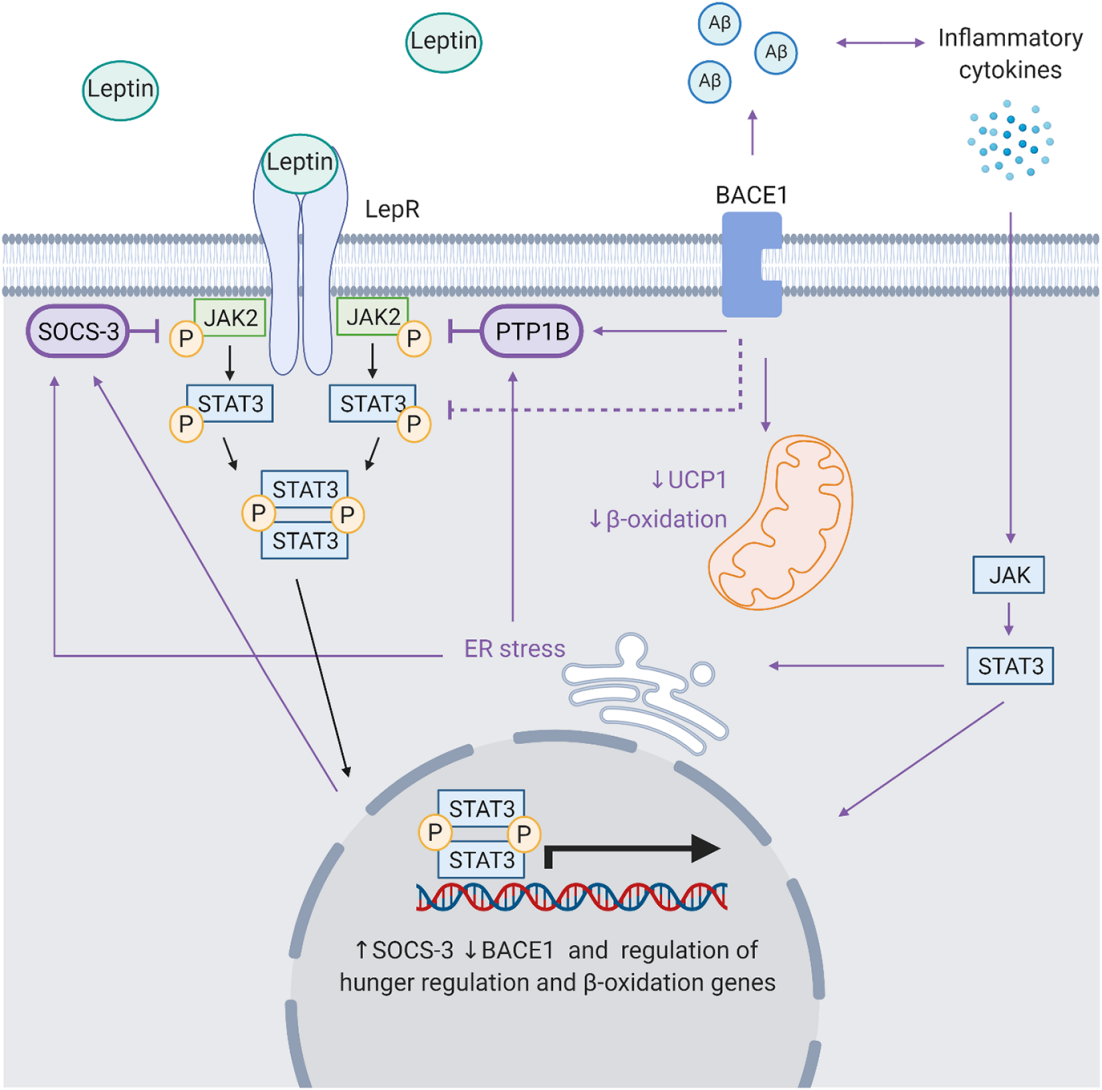
The role of BACE1 in the leptin pathway. BACE1 contributes to dysregulated leptin signaling directly and indirectly. Elevated BACE1 expression is associated with increased PTP1B and SOCS3, which negatively regulate JAK2/STAT3 signaling, preventing leptin‐stimulated gene regulation. When BACE1 is reduced, energy expenditure is increased likely via increases in UCP1 expression and □‐oxidation. Aβ‐mediated inflammation can cause JAK/STAT3 signaling and ER stress, which can increase PTP1B and SOCS‐3 transcription, and regulate genes important in appetite and body weight regulation and β‐oxidation. Created with BioRender.com

In addition to insulin signaling, other metabolic changes associated with BACE1 have been observed, including lowered plasma leptin and restored hypothalamic leptin sensitivity in obese mice.^19^ Leptin is a hormone released by adipocytes, with the principal role to suppress hunger and increase fat breakdown through β‐oxidation.^104^ The leptin receptor (LepR) is localized to the cell membrane, and when bound by leptin, dimerizes, and initiates JAK/STAT signaling (Figure 5). As leptin functions via binding of the leptin receptor, changes in its expression and splicing can reduce metabolism at a given concentration of leptin.^104^ The Janus Kinases (JAK) associated with the LepR phosphorylate the receptor, which in turn phosphorylates signal transducer and activator of transcription 3 (STAT3) proteins. Subsequently, two phosphorylated STAT3 proteins dimerize and bind target genes in the nucleus, leading to a feeling of satiety. Generally, a greater fat deposit in adipocytes results in greater leptin release, and therefore, theoretically, increased lipids should equate to decreased hunger.^104^, ^105^, ^106^

Leptin signaling can also increase lipid breakdown via thermogenesis using β‐oxidation, through innervation of adipocytes by β‐adrenergic receptors.^107^ β‐adrenergic signaling stimulates G protein coupled receptor (GPCR) mediated increase in cAMP production, stimulating PKA, which upregulates the uncoupling protein 1 (UCP1) gene expression. The UCP1 gene encodes an uncoupling protein important in leptin‐induced decreases in white adipose tissue.^108^ In brown adipose tissue (BAT), β‐oxidation produces energy in the electron transport chain, which is given off as heat, thus decreasing the levels of adiposity and maintaining a constant weight.

Impaired leptin signaling is frequently observed in obesity and has been implicated in the development of T2D, with evidence leptin resistance and hyperleptinemia play a pivotal role.^107^, ^109^, ^110^ However, the mechanisms behind this are poorly understood. As plasma leptin levels function as a signal for stored energy levels, the increases in adipose tissue observed in obesity lead to hyperleptinemia (Figure 5B).^106^ This hyperleptinemia is associated with inflammation, hyperglycemia, hyperinsulinemia, insulin resistance and high circulating triglycerides, and an increased risk of atherosclerosis.^111^ In addition to hyperleptinemia, there is evidence that impairments to leptin sensitivity can result from inflammation, including ER stress.^112^, ^113^ There is evidence of an inverse relationship between BACE1 and leptin signaling. BACE1 expression is suppressed by leptin signaling, whereas BACE1 levels are increased by both obesity and T2D.^114^ Therefore, BACE1 may play a role in the mechanisms behind changes in leptin levels and sensitivity.^4^, ^19^ Inhibition of BACE1 in diet‐induced obese (DIO) mice normalized the STAT3 response, alongside improving glucose homeostasis, reducing hypothalamic inflammation, and normalizing hypothalamic leptin sensitivity via NPY, AgRP, and POMC signaling.^19^ Supporting this, BACE1 knock‐in was found to increase adipogenesis, hyperleptinemia, PTP1B, and ER stress.^34^ Interestingly, both BACE1 knockout and inhibitor‐treated mice show no change in daily food intake while presenting with decreased body weight, suggesting body weight changes are likely through increased energy expenditure.^4^, ^19^ This is supported by increased heat production observed in BACE1 knockout mice and is likely a result of the impact of BACE1 on UCP1 expression.^4^, ^115^ By contrast, BACE2 knockout is associated with an adverse metabolic phenotype, including enhanced weight gain, hyperphagia, hyperinsulinemia, and leptin and insulin resistance.^116^ This presents a role for the BACE1 in leptin sensitivity and responsible for energy expenditure and thermogenesis and suggests that the homologous BACE2 enzyme may also play an alternative role.

Elevated levels of circulating fatty acids in the setting of obesity and leptin resistance increase palmitoylation of proteins.^117^ The palmitoylation of BACE1, a modification that has been linked to metabolic dysfunction, namely fatty acid and cholesterol levels, may cause increased BACE1 activity in response to increases in circulating triglycerides. BACE1 is palmitoylated at four cysteines found in C‐terminal cytosolic and transmembrane domains, which consequently stabilizes and increases protein levels.^6^ When palmitoylated, BACE1 localization to lipid rafts is enhanced, promoting Aβ production.^118^ Aberrant palmitoylation in response to metabolic dysfunction has been suggested to extend BACE1 half life, a theory supported by the colocalization of BACE1 and cholesterol in hypercholesterolemia.^119^, ^120^, ^121^ This suggests that altered palmitoylation in response to metabolic dysfunction could be an important mechanism behind pathological increases in BACE1 activity.

Taken together, this suggests BACE1 is a pivotal enzyme in the development of cellular leptin resistance observed in obesity and T2D; however, the mechanism is unclear. Administration of Aβ has been shown to cause leptin resistance (Figure 5).^122^, ^123^ While BACE1‐mediated proteolysis of the leptin receptor is yet to be determined.

## 10 THE ROLE OF BACE1 IN BAT DIFFERENTIATION

**FIGURE 6:**
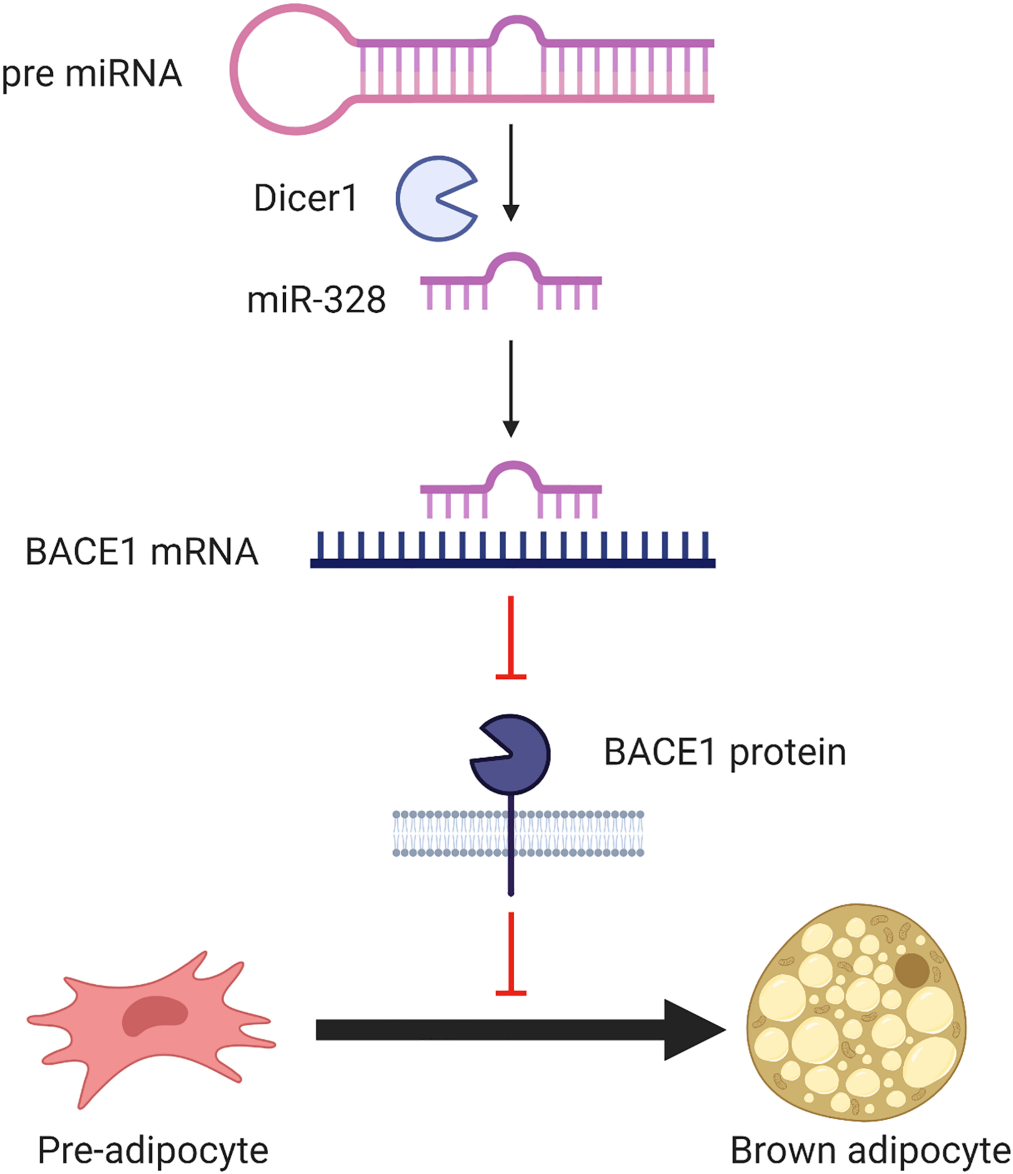
The role of BACE1 in brown adipose fat commitment. Dicer1 cleavage of pre miRNA produces the miR‐328. miR‐328 binds BACE1 mRNA, regulating the stability of the transcript and therefore BACE1 expression. BACE1 promotes myogenesis and consequently inhibits brown adipose tissue (BAT) differentiation. Silencing of BACE1 by miR‐328 therefore increases pre‐adipocyte commitment to brown adipose tissue. Created with BioRender.com

In addition to β‐oxidation via leptin signaling, BACE1 has also been implicated in body weight regulation via thermogenesis and its role in BAT differentiation.^124^ BAT generates heat through lipid breakdown, via UCP1‐mediated mitochondrial uncoupling, a function that has become a therapeutic target for obesity.^125^ The abundance of BAT is greatest in infants and hibernating animals, to protect against hypothermia, as it contains a greater amount of mitochondria than white adipose. BAT dysfunction is associated with dysregulation of glucose metabolism and is observed in aging and metabolic disease.^124^, ^126^, ^127^ This decline in BAT function has been attributed to reduced expression of the microRNA‐processing node, Dicer1.^124^, ^128^ Although the reasons for downregulation of Dicer1 have not been fully elucidated, it has been shown to occur in response to hypoxia.^128^ Dicer1 is an important enzyme in the production of small interfering RNA (siRNA) and microRNA (miRNA), via cleavage of double stranded RNA. Downregulation of Dicer1 leads to reduced expression of functional microRNAs (miRNA). These small non‐coding RNAs regulate stability, degradation and translational ability of target mRNAs and can alter adipocyte differentiation.^129^ In DIO models, Dicer1 downregulation resulted in decreased expression of miR‐328.^124^ This reduction is miR‐328 was accompanied by a decrease in genes associated with BAT function, including UCP1.^124^ The action of miR‐328 in promoting BAT function and differentiation is believed to occur via its silencing of BACE1, preventing BACE1 promotion of myogenesis and therefore inhibition of BAT commitment (Figure 6). Significantly, overexpression of miR‐328 was found to counteract the BAT downregulation seen in obesity.^124^ In addition to the Dicer1/miR‐328/BACE1 axis in BAT function and differentiation, BACE1 overexpression has also been previously linked to reduced miR‐328 activity in the context of AD.^62^ Furthermore, in vivo silencing of BACE1 was found to delay DIO induced weight gain, improve glucose tolerance, increase energy expenditure and insulin sensitivity, and elevate UCP1 expression in brown fat.^4^ This suggests that obesity induced Dicer1 downregulation decreases miR‐328, preventing BACE1 silencing and causing a decrease in BAT differentiation and thermogenesis as well as BACE1‐mediated impairments in glucose metabolism and homeostasis. This presents an important metabolic role for BACE1 and highlights BACE1 as a potential therapeutic target for the treatment of obesity induced diabetes.

## 11 THE EFFECT OF BACE1 ON THE VASCULATURE

BACE1 has been associated with the cardiovascular system via various mechanisms, including angiogenesis, vasculogenesis, vasodilatory pathways, and atherosclerotic plaques. The action of BACE1 has been linked to notch signaling, important in angiogenesis.^130^ BACE1 regulates Notch3 signaling via direct cleavage of JAG1, in turn, increasing aberrant angiogenesis and vessel sprouting.^131^ In addition to the direct action of BACE1, overexpression of Aβ also coincides with a decrease in JAG1/Notch3 signaling.^46^ Although it requires further investigation, it has been suggested that Aβ interact directly with Notch genes.^132^ The injection of Aβ‐42 into the rat hippocampus was found to increase angiogenesis, likely via vascular endothelial factor (VEGF) and an inflammatory response.^133^ However, contradictory studies have shown that both Aβ and BACE1 inhibitors can inhibit angiogenesis in tumor models.^134^ Furthermore, BACE1 is also responsible for ectodomain cleavage of vascular endothelial growth factor receptor 1 (VEGFR1), a receptor important in the regulation of angiogenesis and vascular permeability.^135^ Whether BACE1 can regulate adipose tissue angiogenesis in the setting of metabolic dysfunction is an interesting concept that requires further investigation.

Atherosclerosis is an important factor in the development of cardiovascular disease, including myocardial infarction, cerebrovascular accident, and stroke.^136^ BACE1 is implicated in atherosclerotic plaque formation and is increased in the presence of increased cholesterol. High cholesterol has been shown to affect endocytic trafficking of BACE1 and APP, resulting in increased Aβ production.^137^, ^138^ The production of Aβ is enhanced in atherosclerotic development, with APP knockout mouse models exhibiting a decrease in atherosclerotic plaque formation and increased stability in the aorta.^139^, ^140^, ^141^ BACE1 cleavage of ST6Gal‐1, a protein involved in the terminal step of N‐glycan biosynthesis of glycoproteins, contributes to preventing monocyte transendothelial migration, a pivotal mechanism in the initial stages of atherosclerosis.^142^ ST6Gal‐1 increases adhesion between endothelial cells and monocytes as well as aids monocyte penetration into the endothelial tissue.^17^, ^143^ ST6Gal‐1 is found at decreased levels in atherosclerosis development, this suggests a role for BACE1 in its regulation. Furthermore, the dysregulation of the BACE1/BACE1‐AS/Aβ axis is associated with heart failure, with both BACE1‐AS and BACE1 found upregulated.^38^ This demonstrates the close relationship between BACE1 action, atherosclerosis formation, and cardiovascular disease.

Another impact BACE1 can have on the cardiovascular system is through the Aβ‐induced vascular dysfunction. Aβ binds RAGE (receptor for advanced glycation endproducts) or CD36 receptors, activating NADPH oxidase, leading to production of reactive oxygen species (ROS).^35^, ^36^, ^144^ An excess of ROS causes oxidative stress and decreased expression of endothelial nitric oxide synthase (eNOS).^145^ This results in decreased production of the vasodilatory molecule nitric oxide (NO), and increased endothelin‐1, which impairs the vasodilatory ability of blood vessels.^146^ It has also recently been demonstrated that BACE1 is also modified by the vasodilator NO, which S‐nitrosylates BACE1 at high levels inactivating the enzyme.^147^ Furthermore, accumulation of Aβ in blood vessels, as seen in cerebral amyloid angiopathy (CAA), leads to arterial stiffness.^148^

The action of Aβ on vascular endothelial cells can lead to impairments in tight junctions and BBB dysfunction, capillary degradation, inflammation, impaired vascular clearance, and atherosclerotic plaque formation.^148^, ^149^, ^150^ Together, this presents an important physiological role for BACE1 within the cardiovascular system and as an important enzyme in cardiovascular disease.

## 12 BACE1 IN IMMUNE FUNCTION

AD and cardiometabolic diseases share chronic inflammation and immune cell activation as comorbidities. Immune cells, including T cells and macrophages, important contributors to inflammation, hyperglycemia and obesity‐associated T2D,^151^ have recently been found to be abundant in BACE1 protein suggesting it might play a role in their function.

The inflammation observed in Alzheimer's and cardiometabolic diseases may be both an upstream and downstream effect of BACE1. Nuclear factor‐kappa B (NFκB) is a key regulator of the innate immune system and plays a role in gene expression, inflammation, and oxidative stress.^152^, ^153^ Increases in NFκB activation are associated with obesity,^154^ T2D,^155^ and AD.^152^ NFκB‐associated inflammation has also been experimentally linked to insulin resistance.^156^ The BACE1 promotor has an NFκB binding site. When phosphorylated, NFκB increases BACE1 promotor activity and transcription.^152^ Thus, inflammation induced activation of NFκB, facilitates the upregulation of BACE1 expression, and subsequently increase Aβ production. Activation of NFκB in response to inflammation is observed in macrophages, astrocytes, and microglia and has been implicated in a positive feedback loop in astrocytes whereby increased BACE1 leads to increases levels of neurotoxic Aβ, in turn increasing astrocyte activation and inflammation.^157^ Furthermore, treatment with non‐steroidal anti‐inflammatory drugs (NSAIDs) was found to decrease BACE1 transcription and subsequent production of Aβ.^152^ Therefore, a positive feedback loop of inflammation induced NFκB activation, and increases in BACE1, may be fundamental in cardiometabolic function and disease.

BACE1 has been shown to be important in Th17 differentiation.^158^ BACE1 expression is upregulated in macrophage foam cell regions of atherosclerotic plaques.^141^ In vitro experimentation clarified that the role of BACE1 was predominantly attributed to foam cell development. Foam cells, created through the ox‐LDL activation of macrophages, presented significantly elevated BACE1 levels compared with unstimulated cells. Additionally, once BACE1 was knocked down, the resultant foam cells had significantly less lipid droplets, demonstrating that BACE1 has a role in foam cell differentiation and consequent atherosclerotic plaque formation. Conversely, reduced BACE1 expression in macrophages has been shown to increase macrophage phagocytosis following peripheral nerve injury.^159^ Collectively, this points to BACE1 regulating immune cell function; however, this may be disease and/or stimulus dependent and warrants further investigation.

## 13 ACTION OF METABOLIC DRUGS ON BACE1

The proposed metabolic roles of BACE1 are supported by the common mechanisms and treatment targets between T2D, obesity, and AD, with the use of various antidiabetic and metabolic therapies in the treatment of AD showing promising results.^160^

Although research has proved contradictory, the use of NSAIDs has been tested in the treatment of both AD and T2D.^161^, ^162^, ^163^ NSAIDs function by reducing the disease associated inflammation, via inhibition of NF‐κB signaling; however, they notably also reduce BACE1 transcription.^152^, ^164^ The antidiabetic drug liraglutide, in addition to use in treating T2D, has been investigated for the treatment of AD, obesity, and weight loss.^165^ Liraglutide is a long‐acting glucagon‐like peptide‐1 (GLP)‐1 receptor agonist, which functions via increasing the release of insulin from the pancreas while simultaneously decreasing glucagon release. Liraglutide reduces Aβ plaque production and the severity of AD symptoms.^166^, ^167^, ^168^ The action of liraglutide regarding both Aβ production and alleviating insulin resistance has been shown to occur via reducing BACE1 activity.^169^ Improvements in AD are similarly seen with other antidiabetic drugs, for example Glimepiride, which when used on patients with AD shows improved memory and cognitive functions.^170^ Lixisenatide also shows positive effects on glucose homeostasis and improvement of cognitive functions suggesting a possible therapeutic effect for both AD and T2D.^171^, ^172^ The T2D drug Pioglitazone, a PPAR‐γ agonist, has demonstrated control of plasma Aβ levels, cerebral blood flow, and shown improvements in cognitive function.^173^ This action of Pioglitazone is thought to be through its increasing low‐density lipoprotein receptor‐related protein 1 (LRP1) levels and in turn increasing Aβ clearance.^174^

Metabolic drugs are known to downregulate BACE1, including statins and metformin, and although it requires further investigation, it is possible the desired action of these drugs occurs via their impact on BACE1 expression. The increasing evidence for insulin resistance and glucose metabolism playing an important role in dementia led to the investigation of metformin in AD treatment, where it was found to reduce neuronal insulin resistance and improve glucose uptake via activation of AMPK, IR, and PI3/Akt signaling, and attenuate production of Aβ.^175^ It is important to note other studies have only reported a reduction in Aβ production in response to metformin when in combination with insulin.^176^, ^177^ Significantly, the increased risk of AD seen in patients suffering with T2D is reduced with metformin treatment.^178^ In August 2020 the Metformin in Alzheimer's Dementia Prevention (MAP) study started a multicenter phase 2/3 prevention trial to evaluate the benefit of metformin treatment on AD development. The likely mechanism behind this is the reduction in BACE1 activity in response to metformin treatment.^177^ As insulin and Aβ competitively bind to the IR, reducing BACE1 activity will in turn reduce Aβ and should increase the insulin sensitivity of the IR. As well as presenting BACE1 as an important target in the mechanisms of metabolic drugs, this also highlights insulin insensitivity as an early stage of AD development.^179^ This presents BACE1 as an important molecule in the potential repurposing of metabolic drugs, with the mechanism behind their success arguably dependent on BACE1.

The same could be said for statins, a widely prescribed cholesterol reducing drug. Statins are associated with decreased Aβ formation, attributed to the reduction in cholesterol which in turn regulates BACE1.^180^, ^181^, ^182^, ^183^ Statins also reduce the levels of mevalonate, which normally stimulates cholesterol transporter (apoE) secretion, a risk factor for AD.^184^ A reduction in apoE secretion therefore reduces plaque formation and in turn improves cognitive function.^180^ It is arguable that the metabolic approach to treatment via statins and metformin work primarily via the subsequent downregulation of BACE1. In addition to highlighting the involvement of BACE1 in various physiological functions, this presents BACE1 as an important therapeutic target.

## 14 THERAPEUTIC BACE1 INHIBITION

Owing to its association with the production of Aβ and the development of AD, BACE1 has long been an attractive therapeutic target. Unveiling physiological functions of BACE1 and consequences of its dysfunction, alongside it's established role in AD, highlights the importance of a successful BACE1 inhibitor. Several BACE1 inhibitory drugs have been developed, yet despite progression to phase 3 clinical trials, none have made it to wide clinical use.^185^ This has been attributed to a lack of understanding of the physiological function of BACE1, as highlighted in this review, with the inhibitors either not proving effective or having undesirable effects, including worsening of cognition. However, the BACE1 inhibitors that have been developed have been studied focusing primarily on AD, and arguably side effects deemed undesirable in the treatment of AD may be beneficial in the treatment of metabolic dysfunction. For example, Verubecestat is a BACE1 inhibitor tested in phase 3 clinical trials on patients with mild to moderate AD.^186^ Despite showing a reduction in cerebrospinal fluid Aβ levels to 63%–81%, no beneficial effect on cognition was observed for Verubecestat.^186^ Adverse side effects were seen including an increase in falls and injuries, sleep disturbance, suicidal thoughts, hair color change and weight loss along with decreased appetite.^187^ However, the side effects of weight loss and the impact on appetite deemed negative in the treatment of AD could be hugely beneficial for the treatment of metabolic syndrome. These effects likely occur through the action of BACE1 on the leptin pathway and insulin signaling pathway, and could revolutionize the treatment of metabolic dysfunction. Similar effects were seen with Lanabecestat, another BACE1 inhibitor undergoing clinical trials, with a lack of cognitive improvement observed but with the side effect of weight loss.^188^ A greater number of individuals observed a weight loss of at least 7% when taking Lanabecestat, than individuals taking the placebo, with mean weight loss at −1.9 kg for the 50‐mg Lanabecestat group compared with 0 for the placebo group.^188^ The potential of Lanabecestat in obesity treatment has been recognized by AstraZeneca, who have recently obtained a patent for obesity treatment.^189^ In line with trial findings, the patent highlights differences in weight loss based upon body mass index (BMI), an effect supported by preclinical research.^19^ In trials, weight loss was greatest in individuals with a higher BMI, suggesting Lanabecestat could treat excess weight observed in obesity, without causing extreme weight loss when BMI is reduced. Despite the promise of this approach, it is important to consider the difficulties faced with BACE1 inhibition. The broad expression and substrate profile of BACE1 may be responsible for the various adverse effects on cellular processes observed in clinical trials. However, a greater understanding on the physiological roles of BACE1 may help unravel previous failings. It is also important to note that Verubecestat shows greater selectivity for BACE2 than BACE1, with Lanabecestat having a similar selectivity for both.^190^ It may therefore require further investigation into ensuring these effects are mediated by BACE1. Given the extent of research into effective BACE1 inhibitors for AD, the repurposing of BACE1 inhibitory drugs for treatment of metabolic syndromes such as diabetes and obesity, or as a dual therapy against both diseases, could be a huge advancement in the treatment of metabolic disorders.

## 15 BACE1 AS A BIOMARKER

BACE1 protein or markers of activity have shown promise to be blood‐based biomarkers for a number of diseases, including AD.

BACE1 protein is detectable in plasma and levels are significantly raised in patients with mild cognitive impairment, while also predicting conversion of MCI to AD.^191^ Furthermore, plasma BACE1 protein levels are elevated in people with T2D and correlate with glycemic control, independently of cognition.^192^ The lncRNA species BACE1‐AS strongly correlates with BACE1 expression and is also measurable in human plasma samples, with levels increased in patients with AD^193^ and autism.^194^ Currently, there is a lack of consensus for plasma Aβ as a useful biomarker for AD. However, Aβ levels are elevated in patients with cardiometabolic diseases including obesity, T2D, and heart failure.^38^, ^195^, ^196^

## 16 FUTURE DIRECTIONS

It is clear that the BACE1‐AS/BACE1/Aβ axis has important physiological functions outside the brain. While, elevated BACE1 activity is observed during the development of a number of diseases, in addition to AD. This poses the exciting concept that current BACE1 inhibitors, developed for the treatment of AD, could be repurposed for the treatment of cardiometabolic diseases.

Whether expression and activity of BACE1 could be used as potential prognostic and/or therapeutic biological marker(s) will require further research into disease specificity and sensitivity but remains a promising possibility.

Taken together, this demonstrates the underappreciated functions of an enzyme primarily investigated for its role in AD. BACE1 clearly plays a role in multiple physiological and pathological cellular processes, and future studies are needed to fully understand this important enzyme.

## CONFLICT OF INTEREST

The authors declare no conflicts of interest.
